# “Evaluation of a best practice approach to assess undergraduate clinical skills in Paediatrics**”**

**DOI:** 10.1186/s12909-020-1954-7

**Published:** 2020-02-11

**Authors:** Fabiola Stollar, Bernard Cerutti, Susanne Aujesky, Mathieu Nendaz, Annick Galetto-Lacour

**Affiliations:** 1 Pediatric Department, Children’s Hospital, University Hospitals of Geneva, 6 Rue Willy-Donzé, 1211 Geneva, Switzerland; 2 Unit of Development and Research in Medical Education (UDREM), Faculty of Medicine, University of Geneva, 1 Rue Michel-Servet, 1206 Geneva, Switzerland; 3 Service of General Internal Medicine, University Hospitals of Geneva, 4 Rue Gabrielle-Perret-Gentil, 1205 Geneva, Switzerland; 4 Pediatric Emergency Division, Children’s Hospital, University Hospitals of Geneva, 6 Rue Willy-Donzé, 1211 Geneva, Switzerland

**Keywords:** OSCE, Clinical skills in pediatrics, Medical education, Standardized patients, Assessment method, Standardized grid

## Abstract

**Background:**

The Objective Structured Clinical Examination (OSCE) has been used in pediatrics since the 1980s. Its main drawback is that large numbers of children are needed to make up for the fatigue factor inherent in prolonged testing periods. Also, examinations mainly include children between 7 and 16 years old. We describe the summative examination used in our institution to evaluate medical students’ clinical competencies in pediatrics with realistic available resources and for a wider age-range. We also evaluated different factors known to influence medical students’ performances.

**Methods:**

This retrospective, descriptive, observational study evaluated the 740 distinct pediatric examination results of fourth-year medical students over 5 years. Their summative examination combined two different assessment methods: a structured real-patient examination (SRPE) using standardized assessment grids for the most frequent pediatric diagnoses, and a computer-based written examination (CBWE).

**Results:**

Our approach defined an appropriate setting for some key elements of the educational objectives of pediatrics training, such as balancing the child–parent–pediatrician triangle and the ability to interact with pediatric patients, from newborns to 16-year-old adolescents, in a child-friendly fashion in realistic scenarios. SRPE scores showed no associations with students’ degrees of exposure to specific lecture topics, vignettes, or bedside teaching. The impacts of clinical setting, topic, and individual examiners on SRPE scores was quite limited. Setting explained 1.6%, topic explained 4.5%, and examiner explained 4.7% of the overall variability in SRPE scores.

**Conclusions:**

By combining two different assessment methods, we were able to provide a best-practice approach for assessing clinical skills in Pediatrics over a wide range of real patients.

## Background

Medical education’s primary concern is clinical performance, yet how to measure this consistently remains undefined [1]. Assessment is concerned with «How well the individual performs», [2] more specifically in our study in clinical skills acquisition in pediatrics. The assessment data are used for quality assuring the pass/fail decision, the effectiveness of the clinical performance and the validity and reliability of the tests [3].

Observed long cases can be useful for assessing clinical skills, depending on the sample size of cases and examiners [4, 5]. Modifications to the long case format, by using structured question grids or multiple examiners, [6, 7] have been proposed to find more practicable ways of increasing examination reliability while maintaining the long case’s holistic approach towards the patient, which is part of the attraction of the long case. They have also been recommended for summative assessment [8].

In the 1970s, requirements for more objective tests of clinical skills led to the development of the Objective Structured Clinical Examination (OSCE) [9–11]. This has been used successfully in pediatrics since the 1980s [12]. Some studies have claimed that it is a valid, objective assessment method—more reliable than the short- or long-case clinical examinations used for assessing clinical skills [13]. There are, however, still some concerns and potential drawbacks regarding pediatric OSCEs for medical students. The principal drawback is that these examinations mainly included children between 7 and 16 years old [14]. Other important drawbacks are the time and financial resources required for administering those examinations. The cost of using children as standardized patients (SPs) is about 2.4–3.2 times higher than for using adults [15, 16]. Pediatric SPs are more difficult to use due to the variable physical and psychometric properties inherent in their ages. First, larger numbers of children are necessary to make up for the fatigue factor caused by prolonged testing periods: children may not act reliably and consistently during numerous consecutive examinations, resulting in a less objective, less standardized assessment [14, 17]. Furthermore, it would be impossible to allow a dozen medical students to each spend ten minutes physically examining the same newborn one after the other. Finally, using child SPs is generally more time consuming and complicated than using adult SPs [14].

Different approaches have been taken to avoid the use of real child patients in pediatric OSCE. These include the use of adults simulating parents to assess history taking, [18, 19] the recruitment of healthy primary school-aged children and adolescents as simulated SPs, [11] the use of video recordings of physical findings, microscope slides, photographs of dermatological findings, x-rays, and other forms of imaging [20]. However, these approaches do not allow an assessment of certain key elements of pediatric clinical skills, such as balancing the parent–child–pediatrician triangle and the physical examination of younger children and newborns.

In the assessment of clinical competence it is important to observe a candidate interacting with a patient. The role of the patient in this encounter will vary depending upon the level of interaction expected between the student and the patient, and whether physical signs are part of the presentation [3]. The main reason for involving real child patients is not only testing students’ ability to elicit physical signs, but rather their ability to interact with them in a child-friendly fashion [11].

In light of these difficulties, it seems important to find better-adapted solutions to assess medical students’ clinical competencies in pediatrics over a wide range of real patients [13].

Our institution uses a best practice approach to test clinical skills in pediatrics across a wide age-range, from newborns to adolescents, by combining two different assessment methods: a structured real-patient examination (SRPE) using standardized assessment grids, inspired from structured oral examination, [21] and a computer-based written examination (CBWE). We also created a set of clinical training activities observed by tutors (reported in a portfolio), as a prerequisite to participation in the combined summative examination.

Based on recognized foundations in assessment [22] the present paper describes our evaluation program for medical students’ pediatric clinical skills. It also evaluates the influences of different factors known to affect medical students’ performances.

## Methods

### Study design and subjects

This retrospective, descriptive, observational study examined the results obtained by medical students in their summative pediatrics examinations. These occur during the fourth year of their six-year curriculum, after an eight-week clerkship rotation in the Children’s Hospital of the University Hospitals of Geneva. We assessed the examinations of 740 distinct students over five academic years (2010–2015). To investigate the influence of the chosen examination topic on results, we assessed students’ exposure to each topic in the SRPE. Exposure to the topic via lectures and problem-based learning sessions (vignettes) was recorded from the detailed documented program of the rotation in pediatrics. Exposure via bedside teaching was assessed by having two faculty members classify exposure to each topic as “almost none”, “some”, or “very frequent”. According to a 2009 decision made by the Ethics Committee of Geneva and the University of Geneva’s Faculty of Medicine Teaching Committee Office, research projects in the field of medical education, dealing with existing anonymized data, and designed to evaluate the quality of undergraduate or postgraduate educational programs, are exempt from the need for a full review process and formal approval by the Ethics Committee.

### Description of the portfolio

During their eight-week clerkship in Pediatrics, students attend a series of lectures and workshops covering most of the main pediatric subjects. For clinical activities, they are assigned to a pediatric ward for 3 weeks and spend the remaining five rotating between the pediatric emergency ward, the psychiatric department, and neonatology and specialty outpatient clinics.

Each student must carry out 13 specific clinical activities under the direct supervision of an experienced pediatrician. Students summarize their activities in a portfolio and undergo formative evaluations of their skills in history taking, physical examinations (including of newborns), clinical reasoning, case presentation, and technical procedures in pediatric medicine. If a student is considered inadequate or lacking in certain essential skills, the activity is not validated and must be redone.

The clerkship in pediatrics is considered validated if all the clinical activities were carried out satisfactorily. A validated clerkship is a prerequisite for the student to be able to sit the summative examination in pediatrics.

The use of the portfolio to validate their clerkship as a prerequisite for presenting themselves for the written examination, also allowed the students to see more cases. However, this portfolio is not assessed by a grade participating in the final summative assessment in order to separate the learning part with a formative assessment from the final summative assessment. This procedure is also designed to avoid the comparison of students regarding their level of experience, since the activities do not take place at the same moment in their clerkship.

### Description of the summative examination in pediatrics

The summative examination used to evaluate medical students’ competencies in pediatrics combines two different assessment methods: a structured real-patient examination (SRPE) using standardized assessment grids for the most frequent pediatric diagnoses, and a computer-based written examination (CBWE). The final score for the summative examination in pediatrics is the arithmetic mean of the SRPE and CBWE scores. The conversion scale is adapted for each session annually using the Hofstee scaling method, which also takes into account the performance of the student’s examination group [23].

Students who fail the summative examination (mean final score beneath the pass level) must repeat both examinations (SRPE and CBWE). They are permitted three attempts—failure at the third attempt leads to exclusion from the medical school.

Successful completion of the summative examination in pediatrics is a prerequisite for commencing the sixth year of the curriculum, which is a year spent doing clerkships in several medical disciplines and leading to the Swiss national medical licensing examination.

### Computer-based written examination (CBWE)

The CBWE takes place twice a year: the May session (first session) group’s students who did their clerkships in January/February or March/April, and the January session (second session) for those who did them in May/June, September/October, or November/December. The CBWE uses CAMPUS software provided by the Umbrella Consortium for Assessment Networks (UCAN). This examination tests clinical reasoning skills and the theoretical knowledge learned during rotations in pediatrics. Students are asked to perform step-by-step resolutions of several clinical cases presenting with different common pediatric complaints. Supplemental patient information, given sequentially, allows them to move towards case resolution [24]. The CBWE does not use adaptive computer testing; all students in a particular session face similar questions. Identical supplemental information is given to all students after a step towards the case’s resolution has been validated, independently of the answers given by the students. Answers cannot be changed after the step has been validated. Students have 2 hours to answer 40–45 questions about 10–15 clinical cases. Cases cover general pediatrics, neonatology, pediatric surgery, and pediatric orthopedics. New cases are reviewed by two independent pediatric faculty members. Before new cases are used in examinations, the final version of the CBWE is tested in real-time conditions by a panel of 5–6 experienced pediatricians.

### Structured real-patient examination (SRPE)

The SRPE takes place in the last week of the eight-week clerkship rotation in pediatrics. Each student is evaluated on a single real patient (from a newborn to a 16-year-old adolescent), using a standardized diagnosis-related grid (see Additional file 1: SRPE standardized grid example).

Prior oral consent is obtained from each real patient’s parents/caregivers and from the older children and adolescents. Participants are drawn from three different clinical settings: healthy newborns from the maternity ward, children hospitalized for pediatric, surgical, or orthopedic illnesses, and children coming for follow-up pediatric outpatient clinic visits (non-hospitalized participants receive a gift voucher).

Only cases presenting with a problem listed under the main learning objectives in the Swiss Catalogue of Learning Objectives for Undergraduate Medical Training are used. Examiners are recruited from among Pediatrics Department faculty members actively involved in undergraduate medical teaching. Under the observation of two raters, who remain in the room, students are introduced to a real patient and her/his parents and carry out a focused, structured history-taking and physical examination. Subsequently, after taking leave of the patient, students must summarize their findings to the raters, establish a differential diagnosis, and define a plan of action.

Standardized marking grids have been developed for a list of the 42 most frequent diagnoses made in long-case pediatrics examinations of hospitalized children and outpatient visitors in the 5 years preceding the introduction of the SRPE. The grids were adapted for pediatric settings based on a standardized, validated grid used for OSCE with standardized adult patients. Every grid was reviewed by two experienced faculty members from the Department of General Pediatrics, who evaluated content accuracy and relevance as well as the balance between the ratings of the different grids using objective criteria.

The grids were divided into four parts to evaluate different competencies [25]:

1. History-taking
2. Physical examination
3. Problem-solving (clinical judgment: which includes diagnosis/treatment)
4. Professional attitude/communication skills

Rating is standardized, with the grid indicating the number of points scored for each key element and answer. At the end of each section, additional *general* points can be scored if the student used a structured, focused approach. The *attitudes* section evaluates the overall impression given off by the student, their communication skills, and whether the child and parents were approached in an appropriate and empathetic fashion (see Additional file 1: SRPE standardized grid example). The SRPE takes approximately 90 min. At the end of each SRPE, students receive immediate oral feedback from the raters about their strong points and shortcomings as an additional aid to improving their techniques and learning from the examination. As the SRPE focuses on clinical skills, the history-taking and physical examination sections account for a minimum of two thirds of the possible score.

### Analysis

Results for continuous variables are presented as medians with their interquartile range (IQR). All data were collected in a table by the data management team. SRPE scores were analyzed using descriptive statistics, followed by a mixed-effects model using the following factors: topics (random effect), examiners (random effect), clinical setting, topics available in an e-learning format, topics covered in seminars, and examination session dates. Correlations between CBWE and SRPE scores were also computed. Finally, we included CBWE score, which ought to represent students’ theoretical skill levels, in the model mentioned above. Comparisons of proportions were made using exact binomial tests.

All analyses were made using R software, version 3.2.2 (The R Foundation for Statistical Computing, Vienna, Austria), and TIBCO Spotfire S +® 8.1 for Windows (TIBCO Software Inc., Palo Alto, CA, USA).

## Results

### Structured real-patient examination (SRPE)

The study sample involved the examination results of 740 students (each student had one SRPE and one CBWE). The SRPE results involved the analysis of 42 topics and 56 examiners.

**Fig. 1:**
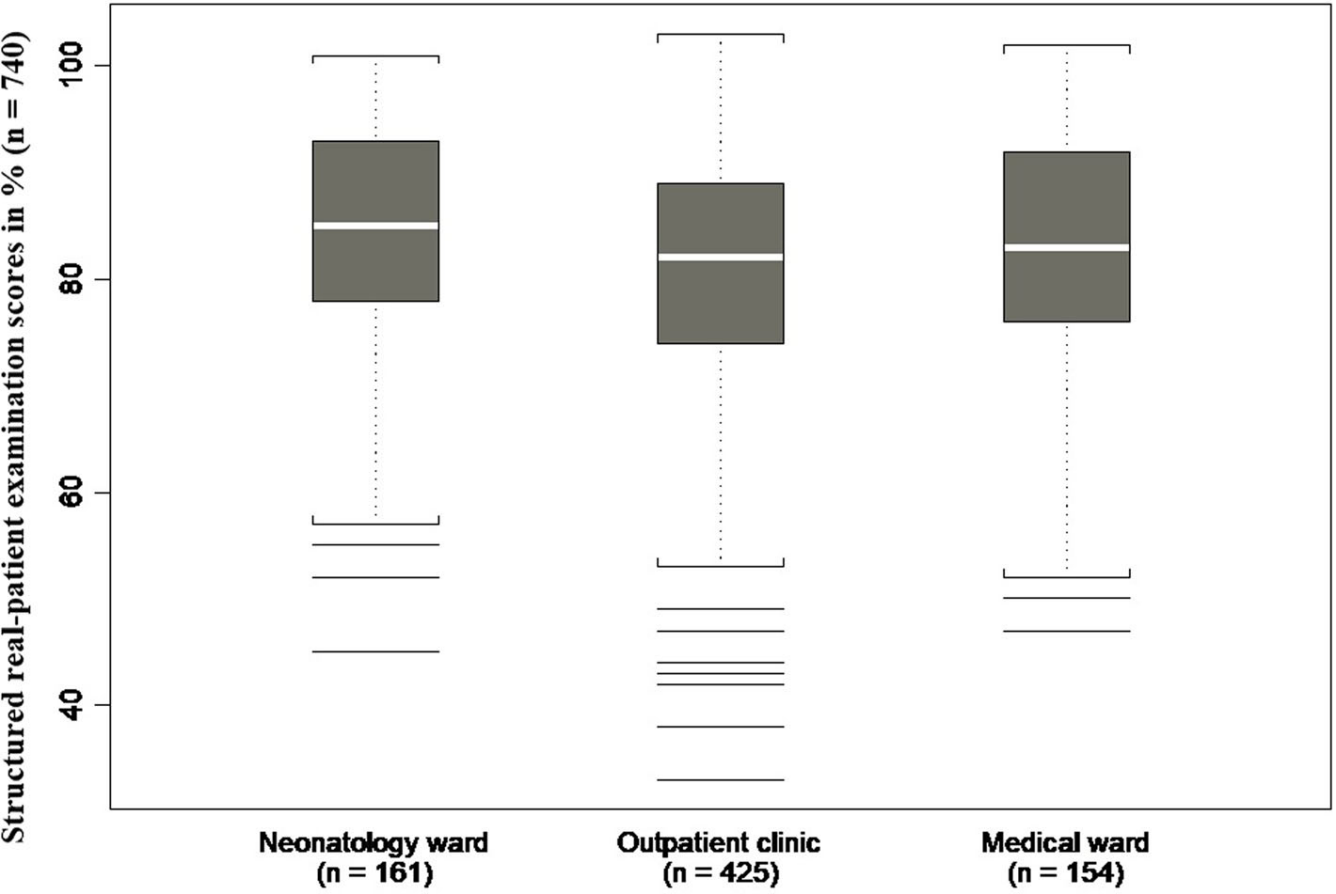
Structured real-patient examination scores depending on clinical setting (*p* = 0.0130)

There were significant differences in scores depending on the topic (*p* = 0.0059), examiner (*p* = 0.0019), and weaker evidence on the clinical setting (*p* = 0.0872; see Fig. 1). There was clearly no evidence of an associations between the scores and the students’ degrees of exposure to specific lecture topics, vignettes (*p* = 0.2259), or bedside teaching (*p* = 0. 99965). The mixed-effects model analysis including the CBWE as an additional factor confirmed the significant effects of the topic (*p* = 0.0029) and the examiner (*p* = 0.0009) on students’ SRPE scores. Topic explained 4.5%, setting explained 1.6%, and examiner explained 4.7% of the overall variability in SRPE scores (Table 1).

**Table 1:** Linear mixed effects model: SRPE score as a function of different potential effect factors

	Without CBWE	*p*-value	With CBWE	*p*-value
	Parameter estimates		Parameter estimates	
Fixed effect	Intercept		Intercept	
Setting^a^				
Outpatient	−2.339	.4387	−2.444	.4249
Medical ward	+ 0.862	.7761	+ 0.933	.7614
Session December^b^	+ 2.746	.0008	+ 1.614	.0487
Topic available in e-learning	+ 1.808	.2259	+ 2.048	.1723
Topic covered in seminar session	−0.008	.9965	+ 0.325	.8603
			Slope	
Score CBWE			+ 0.276	<.0001
Random effects	Standard deviation		Standard deviation	
Topic	2.673	.0059	2.760	.0029
Examiners	2.765	.0019	2.846	.0009

^a^Reference: neonatology ward

^b^Reference: exam session of May

*SRPE* Structured real-patient examination, *CBWE* Computer-based written examination

### Computer-based written examination (CBWE)

**Fig. 2:**
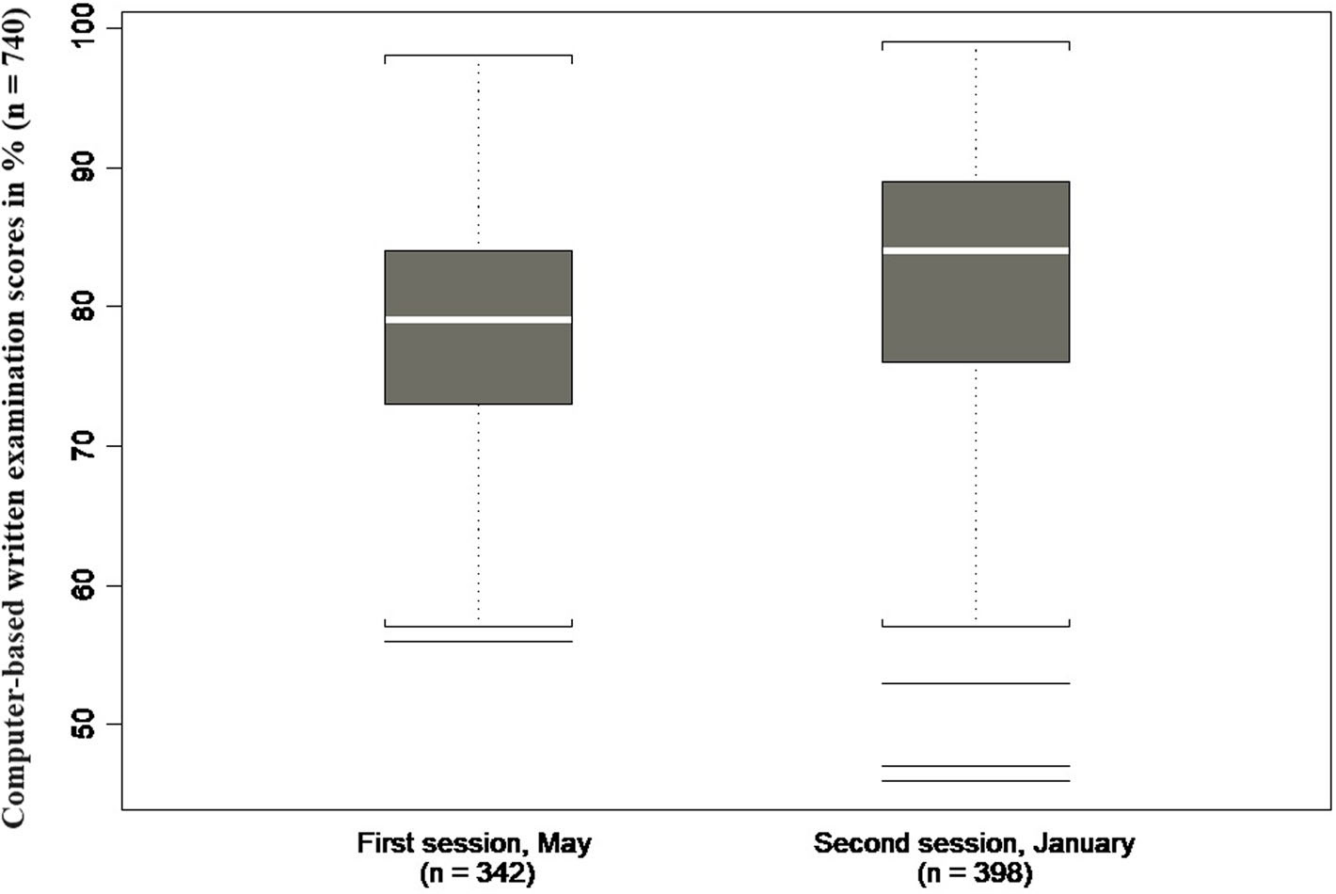
Computer-based written examination scores depending on session date (*p* < 0.0001)

The CBWE was taken by 342 students in May (first session) and 398 in January (second session). Students’ median CBWE scores in the first and second sessions were 79% (IQR: 73–84%) and 84% (IQR: 76–90%), respectively (see Fig. 2). The average Cronbach alpha was 0.716, ranging from 0.578 to 0.796.

Considering the summative examination, there was no significant difference in failure rates between the two sessions (*p* = 0.8685): 4.6% in May and 5.3% in January.

### Correlations between SRPE and CBWE scores

**Fig. 3:**
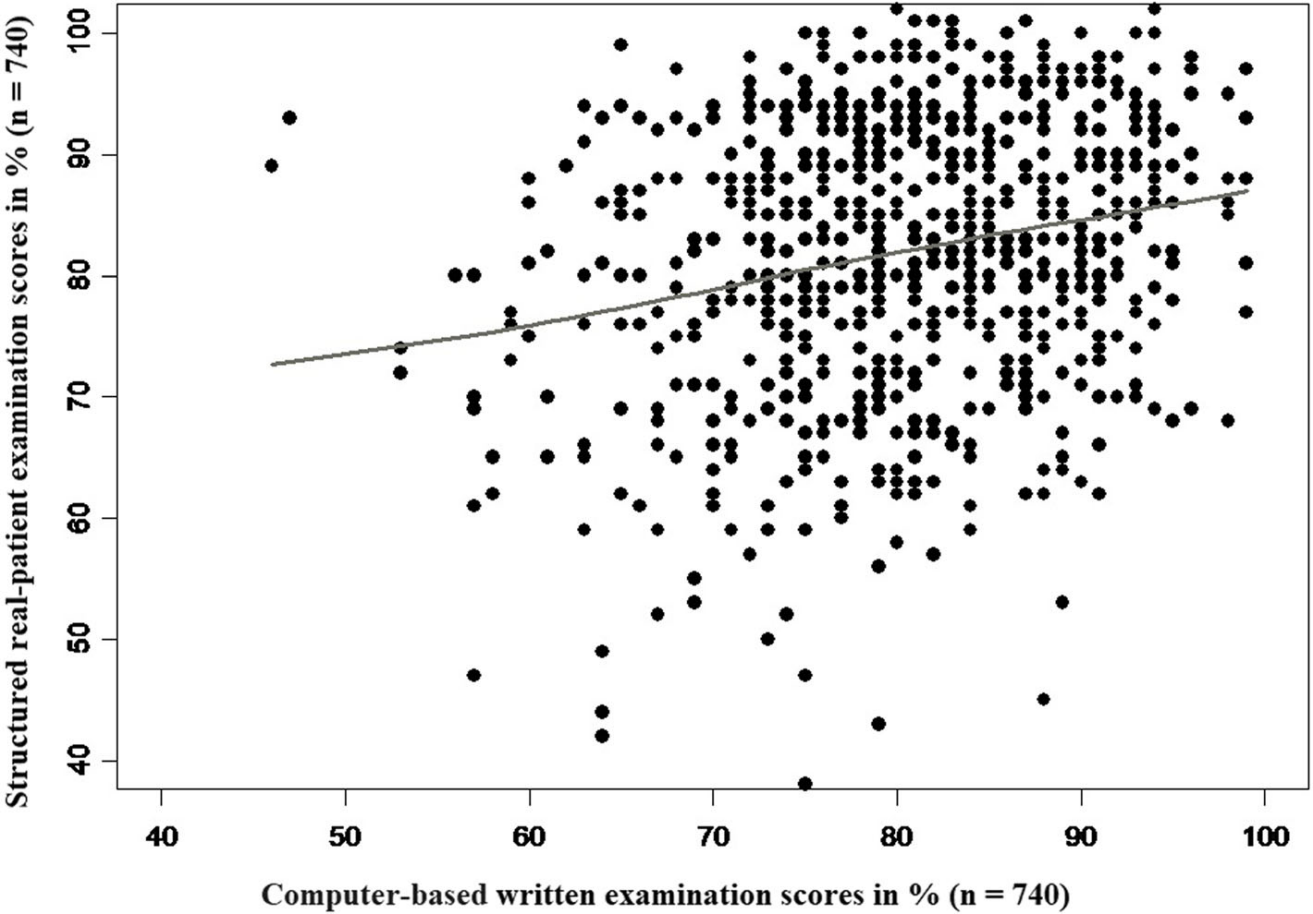
Correlations between structured real-patient examination and computer-based written examination scores (*R*^2^ = 0.051, *p* < 0.001*)*

The median SRPE score was slightly higher than the median CBWE score considering the two sessions (*p* = 0.0019), with 83% (IQR: 75–91%) and 81% (IQR: 75–87%), respectively. The overall median score was 82% (IQR: 76–87%). The correlation between SRPE and CBWE scores was clearly positive but weak (ρ = 0.226; *p* < 0.0001), with only a small proportion of variance explained (*R*^2^ = 5.1%; see Fig. 3).

## Discussion

Our approach, combining the SRPE and the CBWE, enabled us to test various essential educational objectives in pediatrics in real-case clinical settings. These included the ability to make examinations in a child-friendly fashion and the child–parent–pediatrician triangle. The skills of history-taking and clinical examination are fundamental to caring for children, yet they are different from those learned in adult medicine: it is essential that medical students master them [26].

One of the greatest advantages of our approach was that we were able to include pediatric patients of all ages, from newborns to 16-year-old adolescents. Moreover, our set-up was able to develop clinical problems for the most frequent pediatric diagnoses. The SRPE also acts as an encouragement to learning: “I know that I will be tested on my clinical competencies, so I must make an effort to learn them.” This highlights the importance of ensuring that students taking the SRPE have a thorough understanding of the assessment’s purpose, including the benefits of being able to develop and improve their clinical skills based on appropriate feedback.

It is recognized that using a single, long, patient case for examination purposes lacks overall reliability [4, 27]. Even though our SRPE only involved one long case, we tried to compensate for this by creating a summative examination (SRPE + CBWE), such that we were able to increase the number of topics assessed by introducing another 10–15 clinical cases. The use of the portfolio to validate their clerkship as a prerequisite for presenting themselves for the written examination, also allowed the students to see more cases. If we had required the students to pass separately both exams the examination would have been more difficult, but also probably less reliable.

The advantage of incorporating several cases in the pediatric OSCE is that it increases reliability. However, its validity is modest, as some reviews of the pediatric literature relevant to the OSCE have highlighted its drawbacks in addition to the practical difficulties and ethical issues involved in using child SPs [13, 14].

The use of several OSCE stations is hardly possible with preschool children and babies because it implies for children a repetition of stations. The previous publications on pediatric OSCE have mainly included children from 7 to 11 years old and were unable to test all the important clinical competencies required in pediatrics [11, 14]. Moreover, the number of medical students tested in the pediatric OSCEs was lower, the number of stations used was limited (usually three stations), and costs were very high as large numbers of children had to be recruited [14]. Fu et al. described their experience of using SPs for their OSCE. They had to recruit 40 children as each acted as the SP for only three examinees [15]. Furthermore, as some pediatric OSCEs only used older children and adolescents as SPs, authors have claimed that they were forced to use clinical problems dealing with diseases and competencies which apply mainly to adult or adolescent patients [17, 18].

We found that the reliabilities in our CBWE were within the acceptable ranges found in the literature for similar assessments, with a mean Cronbach alpha of 0.71 [28]. Lane et al. reported an inter-station reliability α ranging from 0.64–0.81 in their pediatric OSCE [18]. We were unable to calculate the SRPE reliability as the standardized grids were all different.

A positive but low correlation was found between SRPE and CBWE scores. This supported our hypotheses that each examination tested different aspects of medical students’ competencies. The CBWE allowed us to test students’ knowledge, problem-solving, and patient management for different pediatric topics.

We did not specifically assess the costs of holding the SRPE, a subject beyond the scope of this study. Nonetheless, the SRPE did not generate any particular additional costs, either in personnel or equipment; except for the purchase of a few CHF 50 gift vouchers.

### Factors influencing examination scores

Examiners and examination topics are well known to influence medical students’ scores [29, 30]. Standardized grids seek to neutralize examiner-stringency and help the examination focus on basic questions appropriate for fourth-year students. Our study showed that the impacts of examiners and given topics on overall scores were quite limited; they explained around 4.5% of the overall variability in SRPE scores. We think that the use of standardized grids was important in reducing both differences and impacts.

SRPE scores also depended on the examination’s clinical setting, with better scores obtained in the neonatal unit. This could be explained by the fact that students in our Pediatrics Department are particularly well trained in the physical examination of newborns.

There was clear evidence that students were more likely to get higher scores if their CBWE was taken in the second session (January) rather than in the first (May). This difference was also observed for overall scores and could be explained by the fact that the medical students in the first session have less overall clinical experience. Failure rates did not differ, however. Indeed, using the Hofstee method helped to compensate for differences as it takes into account group success rates.

### Ethical issues

Ethical questions have been raised about involving children in medical students’ examinations, including whether newborns/infants should be examined consecutively by several students [14]. Some authors decided not to use children in their pediatric OSCEs [17] or restricted their choices to children over 7 years old [18]. We decided that each real patient should only be examined by a single student. Another issue is asking parents to consent to the involvement of their children when there is likely no clinical benefit to them. Previous studies have shown that most children and parents said they enjoyed participating in the experience [18, 31]. Before each SRPE, we emphasized to parents that they were not obliged to participate and that *if they* chose *not* to do so, *their child’s* routine medical *care* would in no way suffer. Indeed, after the examinations, we also received positive feedback from children and parents, and we were reassured by our observations of them during examinations. In a previous study, the authors reported that parents felt their children actually learned from the process and would allow them to participate again [18].

### Study limitations

This study had some limitations. First, it had a retrospective design—it would be interesting to collect data prospectively to seek confirmation of our results. Second, we did not specifically assess the feedback from parents, real patients, or students. However, informal feedback from parents and students was overwhelmingly positive. Many parents replied that they and their children enjoyed the SRPE experience. We were also pleased to see outpatients of pediatric subspecialty clinics coming back in consecutive years, looking forward to seeing more students. Most students confirmed that the contents of their summative examinations had been taught during their program. Students also found the SRPE realistic and believed that it was a more appropriate measure of their clinical skills than the CBWE. Moreover, they mentioned the benefits of being able to develop and improve their clinical skills based on appropriate immediate feedback. Third, only one case was used in the SRPE limiting reliability, no controls were included. Lastly, for a best practice approach it would be interesting to make a triangulation with an external qualitative view, as well as an evaluation of students’, faculty members’ and faculty’s view.

## Conclusions

By combining two different assessment methods (SRPE + CBWE), we fostered a best practice approach to testing the essential clinical competencies of medical students faced with large numbers of challenging and varied pediatric cases and topics. These competencies included balancing the child–parent–pediatrician triangle and the ability to interact with patients in a child-friendly fashion, from newborns to 16-year-old adolescents. The use of standardized grids limited the effects of factors known to influence medical students’ scores.

The present study provides medical educators with new options for the assessment of medical students’ clinical skills in pediatrics over a wide range of real patients. The validity of these options should be confirmed in future prospective studies.

## Supplementary information

**Additional file 1:.** SRPE standardized grid example. This file is an example of a standardized grid used in our SRPE.

## Data Availability

The datasets used and analyzed during this study are available from the corresponding author upon reasonable request.

## Abbreviations

CBWE: Computer-based written examination
IQR: Interquartile range
OSCE: Objective Structured Clinical Examination
SPs: Standardized patients
SRPE: Structured real-patient examination
UCAN: Umbrella Consortium for Assessment Networks
