# Supplementary material for: “Evaluation of a best practice approach to assess undergraduate clinical skills in Paediatrics”
Source: BMC Med Educ. 2020 Feb 11;20:46. doi: 10.1186/s12909-020-1954-7 (PMC7014729; doi:10.1186/s12909-020-1954-7)
Supplement: Supplementary file 1 — Additional file 1:. SRPE standardized grid example. This file is an example of a standardized grid used in our SRPE. [file 12909_2020_1954_MOESM1_ESM.pdf]

Student name:

A) History

Date:

For each of the following items, indicate the elements the student was able to specify during history taking (yes/no).

Questions	yes	no
The student specified :		
- *frequency and aspect of vomiting*	1	0
- *frequency and aspect of stools*	1	0
- *duration of complaints*	1	0
- *temperature /fever*	1	0
The student specified :		
- *liquid /water intake*	1	0
- *urine output*	1	0
- *weight loss*	1	0
- *general condition and level of consciousness (does he seem tired? Does he still play?)*	1	0
The student looked for signs of other illnesses associated with vomiting :		
- *headache and neck pain*	1	0
- *burning or frequent urination*	1	0
- *cough/ nasal discharge*	1	0
- *localized abdominal pain*	1	0
The student tried to specify the origin of the gastroenteritis:		
- *infectious contacts (family, school, etc)*	0.5	0
- *recent traveling*	0.5	0
- *food intake*	0.5	0
- *antibiotics*	0.5	0
The student took a personal history (with immunization)	1	0
History taking progressed in a structured and logical way	Excellent: 2<br>Correct: 1<br>Insufficient: 0<br>Inadequate: -1<br>Very inadequate : -2	
The questions were focused on the patients complaints and problems	Excellent: 2<br>Correct: 1<br>Insufficient: 0<br>Inadequate: -1<br>Very inadequate : -2	

B) Physical Examination

The student has to describe what he does and what he is looking for.

Questions	yes	no
The student judged general condition and level of consciousness **(2 points if complete, 1 point if incomplete)**<br>- vital signs<br>- level of consciousness	2 1	0
The student looked for signs of dehydration :		
- *dry mouth and lips*	1	0
- *weight loss*	1	0
- *crying without tears*	1	0
- *decreased skin turgor*	1	0
- *sunken eyes*	1	0
- *vital signs*	1	0
- *sunken fontanelle*	1	0
The student looked for meningitis signs :<br>*> 1 year old : - stiff neck*<br>*- Kernig/Brudzinski signs*<br>*< 1 year old : - bulging fontanelle*<br>*- hypotonia*	2 1	0
The student performed an abdominal examination :		
- *palpation (liver, spleen, abnormal mass)*	1	0
- *rebound tenderness/ involuntary guarding*	1	0
- *localization of the pain*	1	0
- *auscultation of bowel sounds*	1	0
The elements of the physical examination were focused on the patients problem and complaints	Excellent: 2<br>Correct: 1<br>Insufficient: 0<br>Inadequate: -1<br>Very inadequate: -2	
The physical exam followed a logical structure and was adapted to the child's age	Excellent: 2<br>Correct: 1<br>Insufficient: 0<br>Inadequate: -1<br>Very inadequate : -2	

Student name:

C) Diagnosis and treatment

Diagnosis	yes	no
1) Case summary<br><br>Case summary was clear, organized and followed a logical path	Excellent: 2<br>Correct: 1<br>Insufficient: 0<br>Inadequate: -1<br>Very inadequate -2	
2) Differential diagnosis<br><br>Differential diagnosis focused on the most frequent and most likely illnesses	Excellent: 2<br>Correct: 1<br>Insufficient: 0<br>Inadequate: -1<br>Very inadequate : -2	
The student found the 3 main illnesses associated with fever and vomiting :		
- *gastroenteritis*	1	0
- *urinary infection*	1	0
- *meningitis*	1	0
<u>3) Diagnosis :</u><br>The student found the correct diagnosis *: Gastroenteritis*	1	0
<u>4) Knowledge question</u><br>The student knows the main etiology responsible for acquired gastroenteritis in Switzerland:		
- *70% viral*	1	
- *Rotavirus*	0.5	0
- *Adenovirus, Norovirus (0.5 point if finds at least 1 virus)*	0.5	0
- *Salmonella*	0.5	0
- *Campylobacter*	0.5	0

C) Diagnosis and treatment (cont.)

Management	yes	no
<u>1) laboratory investigations:</u><br>They were focused on the problem	Excellent: 1<br>Correct: 0.5<br>Insufficient: 0<br>Inadequate: - 0.5<br>Very inadequate: - 1	
The student knows the main laboratory investigations indicated for this case :<br>*Essential :*<br>- *Blood gas*<br>- *Osmolality (serum and urine)*<br><br>(***2 points if finds all items, 1 point if incomplete)***<br>--<br>*Possible :*<br>- *stool analysis (blood and white blood cells)*<br>- *stool culture*<br>- *blood culture*<br>- *virus detection in the stool*<br>- *Na , K (serum)*<br>- *blood cell count, CRP*<br><br>***(1 point if find at least 2 exams)***	2 1<br>-----------<br>1	0<br>------------<br>0
<u>2) Therapy :</u><br>The student knows the first goal and priority : *rehydration*<br><br>The student knows the 2 possible approaches to rehydration and can calculate the correct amount of fluid replacement :<br><br>*Oral :*<br>- *oral rehydration solution (water , electrolytes, sugar) in fractioned small amounts 50 ml/kg/4 hours*<br>- *nasogastric tube*<br>*Intravenous :*<br>- *calculate the amount by 24 h : normal daily fluid requirements, replacement of deficit and current losses*<br><br>***(1 point if complete and correct answer, otherwise 0 points)***	1<br><br>1<br><br>1	0<br><br>0<br><br>0
**Open question of General Pediatrics :**<br>…………………………………………………………………………………….<br>Suggestions :<br>- How would you evaluate the psychomotor development of this child? (infant or toddler)<br>- Which questions would you use to asses physical and mental health of adolescent patients? (Heeadsss)<br>- Other questions: immunization, infant feeding guidelines, etc	4 3 2 1	0

Student name:

D) Attitude

	yes	sometimes	no
The student started by :			
- greeting the parents (the child)	0		-1
- presenting himself (name, function)	1		0
- trying to establish contact with the child	0		-1
- the student started history taking with an open question	1		0
During the interaction with the parents/the child :			
- the student used plain language (no medical expressions)	1	0	-1
- the student was always adequate (behavior, appearance, body language)	0	-0.5	-1
- the student is attentive towards the parents (listens, doesn't interrupt, tries to understand their fears)	0	-0.5	-1
- the student is attentive towards the child<br>○ **infant** : looks at him and tries to establish contact<br>○ **child** : lets him take part in the discussion, listens to him and uses a language he can understand<br>○ **adolescent** : interacts directly with him, not through the parents	1	0	-1
During physical examination :			
- the student uses an age-adapted approach	0	-0.5	-1
- the student explains what he is doing	1	0.5	0
- the student respects the fears of the small child/ the privacy of the older child	0	-0.5	-1

Student name:

Final result sheet Date:

Standard exam:

Part	Student Score	Maximal Score
A) History<br>B) Physical examination<br>C) Diagnosis and treatment<br>D) Attitude	……<br>……<br>……<br>……	19<br>19<br>22<br>5
Total<br>Percentage	……<br>……	65<br>100%

Name and signature of the experts: 1)

2)

This part will be filled out by the committee:

Score: /65 = %
